# The relative age effect in Brazilian national basketball teams over time

**DOI:** 10.3389/fspor.2025.1716826

**Published:** 2026-01-06

**Authors:** Matheus Neves Rufino Pereira, Francisco Zacaron Werneck, Gabriel Torres da Silva, Hélder Zimmermann de Oliveira, Lívia Costa dos Reis Souza, Pedro Silva Carvalho, Davi Romeiro Soares Alves, Dilson Borges Ribeiro Junior

**Affiliations:** 1 Faculty of Physical Education and Sports, Postgraduate Program in Physical Education, Federal University of Juiz de Fora (UFJF)/Federal University of Viçosa (UFV), Juiz de Fora, Minas Gerais, Brazil; 2 Facult of Physical Education, Salgado de Oliveira University, Juiz de Fora, Minas Gerais, Brazil

**Keywords:** sports talent, talent identification, team, young athletes, elite athletes

## Abstract

**Introduction:**

In basketball, identifying and developing potential athletes is crucial, and the Relative Age Effect (RAE) plays an important role in this process. However, could this effect interfere in the selection of elite players?

**Objective:**

The aim of the present study was to evaluate the RAE in Brazilian male and female basketball national teams in official competitions and to examine its association with the competition type, age category and collective performance.

**Methods:**

A total of 1,585 athletes were analyzed, including 703 males and 882 females. The mean age of the male athletes was 20.99 years old (±5.80), and their mean height was 196.72 cm (±9.36), while the female athletes had a mean age of 20.83 years old (±5.80) and a mean height of 179.00 cm (±6.44). The sample consisted of athletes who participated in world and continental competitions between 2002 and 2023, in the U15, U16, U17, U18, U19 and senior categories. The data were collected from the International Basketball Federation's website (https://www.fiba.basketball). Athletes' months of birth were categorized into quartiles and semesters, and the medalists and non-medalists team performance was analyzed, considering world and continental competitions besides youth vs. senior categories. To investigate the RAE across categories, the Chi-Square (*χ*^2^) test for quartiles was applied. To analyze the association of the RAE (semester) with competition type, outcome and category, the Chi-Square (*χ*^2^) test was used as well.

**Results:**

The findings revealed a significant presence of the RAE (*p* < 0.001) across all groups analyzed, regardless of gender, competition type, category or team performance. No associations were observed between semester of birth and competition type or team performance for either gender. Regarding category type, a significant association was found between being born in the first semester and participating in youth competitions for males (*p* = 0.03) and females (*p* = 0.003).

**Conclusion:**

The RAE is present at the elite level of Brazilian basketball over time, showing association only with the type of category.

## Introduction

Basketball, as a global cultural phenomenon, has been steadily rising in popularity within society. This increase is evidenced by its growing media exposure, widening access to sports practice, and higher occurrence of its events. As a result, an ever-growing contingent of children and youth across various ages have become involved with basketball, whether as competitive practice, recreational engagement, or simply as spectators in search of entertainment (1, 2). In basketball, as in any other team sport, the process of selecting and identifying potential talents is a study field which aims at verifying the factors that determine athletic development from the initial stages to specialization (3). The understanding and the identification of variables that contribute to sports success can facilitate the planning of training and the competitive selection of athletes, optimizing the use of time and resources invested in the sport discipline (4).

For Cobley et al. (5), talent is the understanding of performance with future sports potential. Thus, an athlete who currently excels and demonstrates potential for a promising future, with multiple skills developed over time, is classified as a sport talent (6). According to Fransen and Gullich (7), to identify and select sports talents, it is necessary to observe the dynamic interaction among the individual aspects, the task and the environment. Therefore, based on the theoretical conception of talent centered on the dynamic systems approach, it is important to perceive talent emerging from the interaction of these three factors (individual, task and environment) (7–9).

Among the individual-related factors, it is possible to highlight the Relative Age Effect (RAE). When there is, in a sample of athlete individuals, an overlap of those born in the first months of the year, there is the RAE (10). The RAE is more observed in sports where strength, power and body size are determinants for good performance (11).

Within the most common organizational model of youth categories, in the age groupings in every two years, for example, U-12, U-14, and U-16, an individual can compete with another athlete up to 23 months older, that is, two individuals who may be in completely different maturational stages (12). Consequently, this difference can prioritize chronologically older individuals, especially during the puberty period (13). Thus, young athletes who present a chronological advantage, even if temporary, tend to be chosen more often (10, 14).

In a study conducted with youth athletes in France from U-7 to U-18, RAE was observed in all male and female categories (15). RAE was also found in the FIBA World Championships from 1979 to 2011 in the U-17 and U-19 categories for both genders (16). In the study by Vegara-Ferri et al. (17), which analyzed the RAE in the U-17 and U-19 basketball world championships and in the basketball teams participating in the Rio de Janeiro Olympic Games, it was verified the RAE in the U-17 and U-19 categories. Additionally, the RAE was observed in the group of teams with the best placement in both genders in all competitions considered in the sample, however, the RAE was not observed in the teams with the worst placement in both genders. In another investigation, now with the basketball teams participating in the 2012 Olympic Games, it was found that only the French men's team presented the RAE; therefore, there was no RAE in the Brazilian team in either gender (18). In another analysis, in a study with female athletes from the first and second divisions of Italian basketball, it was possible to observe the RAE (19).

The RAE in Brazilian men's basketball competitions is widely debated, the sample of young basketball players who competed in the 2015 U-17 and U-15 Brazilian Championships showed RAE in both categories (20, 21). In the investigation by Ribeiro Junior et al. (22), RAE was observed in the male categories under 12, 13, 14, and 16 in the Brazilian basketball teams championships in 2017. Furthermore, in a 15-year period (2004–2015), the RAE was found in the Brazilian championships for the U-15 and U-17 categories, in the Basketball Development League (LDB) which includes athletes under 22, and in the Novo Basquete Brasil (NBB), the main adult competition in the country (23).

In Brazilian basketball the RAE in women's competitions was investigated by Coutinho et al. (24), a study that analyzed the group of athletes who competed in the Women's Basketball League (LBF) in the 2011–2012 season and the Brazilian championships nationals in the U-15 and U-17 categories in 2011. In this research, it was possible to observe the RAE only in the U-15 category. In another investigation, involving female adult athletes who participated in the LBF, but in the 2014–2015 season, it was not possible to observe the presence of RAE in this group, in agreement with the study mentioned previously (14). Moreover, in the investigations by Oliveira et al. (20, 21), which examined the presence of RAE in the Brazilian women's basketball championships for the U-15 and U-17 categories in the year 2015, respectively, RAE was only observed in the U-17 category.

Brazil occupies a prominent position in both the global and continental basketball landscape, with athletes playing in the world's top leagues and who have achieved significant results throughout its history (12). Although Brazil has athletes competing in the leading basketball leagues worldwide, its process of identifying and selecting young athletes depends on isolated actions, in the absence of a coordinated national or regional process capable of better harnessing this sports potential. However, considering the presence of RAE in basketball and Brazil's entire sports legacy, it is perceived the need for investigations into the influences of RAE on Brazilian national teams over time.

Therefore, it is noted that the RAE is present in various forms in Brazilian basketball. Yet, does this effect interfere in the selection process of elite athletes, for those who become part of the Brazilian national teams? Moreover, does the RAE persist over time across different competitions? And even, is there an RAE in the group of individuals who were victorious in the national team? Thus, in order to answer the aforementioned questions, the aim of the present study was to evaluate the RAE in Brazilian basketball national teams in both genders in official competitions and to observe its association with competition type, age category, and outcome.

## Methods

### Study design

This study has a retrospective design with a longitudinal analysis, observing the Brazilian youth and senior basketball national teams in world competitions (involving competitions with representatives from all continents) and continental competitions (involving competitions with representatives from the American continent) over 22 years (2002–2023), the characteristics of the RAE, and its relationships with competition type, age category, and outcome (25, 26).

### Sample

A total of 1,585 Brazilian-born basketball players were observed—only players that were born in Brazil, consisting of 703 males and 882 females, who competed in world and continental competitions between 2002 and 2023, representing the Brazilian national team. The continental competitions observed were the South American Championship and the America's Cup, and the world competitions observed were the World Cup and the Olympic Games. The South American Championship includes athletes from the U-15, U-17, U-18 and senior categories. The America's Cup competition features athletes from the U-16, U-18 and senior categories. The World Cup includes athletes from the U-17, U-19, and senior categories. Lastly, the Olympic Games only include senior athletes.

### Procedures

Athlete information (date of birth and team collective performance) were collected from the International Basketball Federation (FIBA) website (https://www.fiba.basketball). The use of the publicly available data from the internet for RAE analysis has been described in other studies without requiring ethics committee approval (18, 27). The use of publicly accessible information is authorized without requiring evaluation by the Ethics and Research Committee (CEP), according to Resolution CNS No. 510, April 7, 2016, from the Brazilian Ministry of Health.

To analyze the data, each player's birth month was categorized into quartiles: the 1st quartile (Q1) comprised athletes born between January 1st and March 31st; the 2nd quartile (Q2), athletes born between April 1st and June 30th; the 3rd quartile (Q3), athletes born between July 1st and September 30th; and the 4th quartile (Q4), athletes born between October 1st and December 31st. Additionally, athletes' birth dates were stratified by birth semester: 1st semester (H1), for athletes born between January 1st and June 30th, and 2nd semester (H2), for athletes born between July 1st and December 31st. An equitable distribution between quartiles and semesters was assumed, based on the reference population of live births in Brazil between 1965 and 2005, from Information System on Live Births (SINASC), Ministry of Health (http://datasus.saude.gov.br).

For the analysis of the association between RAE and the performance achieved by the Brazilian National Team, athletes were classified as medalists (team ranked in the top three places in the competition) or non-medalists (other placements). To analyze the competition type, it was considered world and continental competitions. Finally, to analyze the age category, the sample was divided into youth (U-15 until U-19) and senior categories.

### Data analysis

Statistical analysis was performed descriptively to characterize the sample, using mean ± standard deviation for quantitative variables. For the distribution of birth quartiles and semesters, a descriptive analysis of frequency and percentages was conducted for qualitative variables. To investigate the RAE in the evaluated categories, the Chi-Square test (*χ*^2^) was used for quartiles, and the Odds Ratio (OR) with a 95% confidence interval (CI) was calculated. The OR compared the distribution of the first three birth quartiles (Q1, Q2, and Q3) with the last quartile (Q4), following the recommendations of Cobley et al. (11). In order to verify the association of the RAE (semester) with the competition types, category, and collective performance, a bivariate analysis was performed using a cross-tabulation table with Pearson's Chi-Square test (*χ*^2^), calculating the Odds Ratio (OR) with a 95% confidence interval (CI) between semesters. The effect size was interpreted as follows: OR < 1.23 (very small), OR between 1.23 and 1.85 (small), OR between 1.86 and 2.99 (medium), and OR > 2.99 (large) (28). The data were analyzed using the SPSS statistical software, version 24.0 (IBM Corp., Armonk, NY), with a statistical significance set at *p* < 0.05.

## Results

The sample consists of 1,585 athletes, 703 male and 882 female. For the descriptive analysis of the sample, the mean age and mean height were considered. In Table 1, the mean age and height are presented.

**Table 1:** Mean and standard deviation of age (years) and height (cm) for the male and female group.

Variables	Male		Female	
	Age (years)	Height (cm)	Age (years)	Height (cm)
Total	20.99 (±5.80)	196.72 (±9.36)	20.83 (±5.80)	179.00 (±6.44)
Senior	26.85 (±4.63)	199.20 (±9.22)	27.36 (±4.82)	182.36 (±9.25)
Youth	16.93 (±1.28)	194.45 (±8.93)	16.82 (±1.31)	179.14 (±8.07)
World	25.32 (±6.12)	198.10 (±9.58)	22.47 (±5.82)	181.64 (±8.76)
Continental	19.88 (±5.15)	196.10 (±9.22)	20.44 (±6.05)	180.22 (±8.77)
Medalist	19.86 (±5.03)	198.55 (±9.35)	20.62 (±6.07)	180.32 (±8.88)
Non-Medalist	22.41 (±6.37)	196.90 (±9.40)	21.64 (±5.97)	181.22 (±8.59)

Tables 2, 3 display the results of the birth date distribution of male and female athletes, respectively, into quartiles based on the total sample, competition type, age category, and performance, presenting the values for the *χ*^2^ test, p, and OR. Regarding the male athletes group (Table 2), significant results were found in all comparisons, with the highest OR for the youth category (Q1 × Q4—4.09). Additionally, it is noteworthy that in the Q1 × Q4 comparison, across all male groups, no OR was considered very small or small. Regarding the female group (Table 3), significant results were found in all comparisons, with a “large” effect size OR for the youth category (Q1 × Q4—3.13).

**Table 2:** Male—evaluation of birth quartiles by competition type, age category, and collective performance between 2002 and 2023 for Brazilian national teams.

Competition	*N*	Number (%) of athletes per quartile				*X* ^2^	*p*	OR (95% confidence interval)		
		Q1 (%)	Q2 (%)	Q3 (%)	Q4 (%)			Q1 × Q4	Q2 × Q4	Q3 × Q4
Total	703	258 (36.7)	213 (30.3)	160 (22.8)	72 (10.2)	109.04	<0.001*	3.58 (2.56–5.01)	2.95 (2.10–4.15)	2.22 (1.56–3.14)
World	144	42 (29.1)	51 (35.4)	35 (24.3)	16 (11.2)	18.34	<0.001*	2.62 (1.25–5.50)	3.18 (1.54–6.60)	2.18 (1.03–4.63)
Continental	559	1,256 (36.5)	958 (27.9)	702 (20.4)	523 (15.2)	96.90	<0.001*	2.40 (1.85–3.10)	1.83 (1.41–2.36)	1.34 (1.03–1.74)
Youth	415	184 (44.3)	107 (25.7)	79 (19.0)	45 (11.0)	101.35	<0.001*	4.09 (2.67–6.25)	2.37 (1.52–3.70)	1.75 (1.11–2.77)
Senior	288	74 (25.7)	106 (36.8)	81 (28.1)	27 (9.4)	45.36	<0.001*	2.74 (1.58–4.74)	3.92 (2.30–6.70)	3.00 (1.74–5.17)
Medallist	392	151 (38.5)	111 (28.4)	89 (22.7)	41 (10.4)	64.36	<0.001*	3.68 (2.36–5.74)	2.70 (1.71–4.26)	2.17 (1.36–3.45)
Non-Medalist	311	107 (34.5)	102 (32.8)	71 (22.8)	31 (9.9)	47.26	<0.001*	3.45 (2.07–5.73)	3.29 (1.97–5.48)	2.29 (1.35–3.87)

Source: The authors.

*χ*^2^, Chi-square test; Q1, Jan–Mar; Q2, Apr–Jun; Q3, Jul–Sep; Q4, Oct–Dec; Effect Size, OR <1.23 (very small), OR between 1.23 and 1.85 (small), OR between 1.86 and 2.99 (medium) and OR > 2.99 (large).

* *p* < 0.05.

**Table 3:** Female—evaluation of birth quartiles by competition type, age category, and collective performance between 2002 and 2023 for Brazilian national teams.

Competition	*N*	Number (%) of athletes per quartile				*X* ^2^	*p*	OR (95% confidence interval)		
		Q1 (%)	Q2 (%)	Q3 (%)	Q4 (%)			Q1 × Q4	Q2 × Q4	Q3 × Q4
Total	882	347 (33)	189 (21.4)	167 (19)	179 (23)	97.86	<0.001*	1.93 (1.58–27)	1.05 (9.84–12)	0.93 (0.74–1.17)
World	227	88 (38.7)	40 (17.6)	38 (16.7)	61 (26.8)	28.66	<0.001*	1.44 (0.99–2.)	0.65 (0.42–11)	0.62 (0.39–07)
Continental	655	259 (35)	149 (22.7)	129 (19.6)	118 (11)	76.89	<0.001*	2.19 (1.72–29)	1.26 (0.96–14)	1.09 (0.83–15)
Youth	535	219 (49)	127 (23.7)	119 (22.2)	70 (13.0)	86.69	<0.001*	3.13 (2.33–40)	1.81 (1.32–28)	1.70 (1.23–23)
Senior	347	128 (38)	62 (17.8)	48 (13.8)	109 (34)	49.69	<0.001*	1.17 (0.87–17)	0.57 (0.40–00)	0.44 (0.30–03)
Medallist	584	230 (33)	138 (23.6)	106 (11)	110 (18)	68.60	<0.001*	2.10 (1.52–20)	1.25 (0.95–15)	0.96 (0.72–18)
Non-Medalist	298	117 (32)	51 (17.1)	61 (20.4)	69 (23.1)	34.51	<0.001*	1.70 (1.20–27)	0.74 (0.50–10)	0.88 (0.60–19)

Source: The authors.

*χ*^2^, Chi-square test; Q1, Jan–Mar; Q2, Apr–Jun; Q3, Jul–Sep; Q4, Oct–Dec; Effect Size, OR <1.23 (very small), OR between 1.23 and 1.85 (small), OR between 1.86 and 2.99 (medium) and OR > 2.99 (large).

* *p* < 0.05.

Tables 4–6 present the distribution of birth semesters in relation to competition type, category and outcome, respectively. In Table 5, the results indicate an association between semesters and category type for both genders. For males, *p* = 0.03 with an OR indicating a small effect (1.41), and for females, *p* = 0.003 with an OR also indicating a small effect (1.51). No significant associations were found between type of competition and performance for either gender (Tables 4, 6).

**Table 4:** Evaluation of birth semesters of athletes participating in Brazilian national teams between 2002 and 2023: competition type.

Variable	Type of competition Men			*X* ^2^	*p*	OR (95% confidence interval)	
	World	Continental	Total			1st semester × 2nd semester	Effect size
Semester							
1st semester	93 (19.7)	378 (80.3)	471				
				0.48	0.49	0.873 (0.59–1.28)	Very small
2nd semester	51 (22.0)	181 (78.0)	232				

Variable	Type of competition Woman			*X* ^2^	*p*	OR (95% confidence interval)	
	World	Continental	Total			1st semester × 2nd semester	Effect size
Semester							
1st semester	128 (23.9)	408 (76.1)	536				
				2.46	0.117	0.783 (0.57–1.06)	Very small
2nd semester	99 (28.6)	247 (71.4)	346				

Source: The authors.

*χ*^2^, Chi-square test; H1, Jan–Jun; H2, Jul–Dec; Effect Size, OR <1.23 (very small), OR between 1.23 and 1.85 (small), OR between 1.86 and 2.99 (medium) and OR > 2.99 (large).

*p* < 0.05.

**Table 5:** Evaluation of birth semesters of athletes participating in Brazilian national teams between 2002 and 2023: age category.

Variable	Category type Men			*X* ^2^	*p*	OR (95% confidence interval)	
	Youth	Senior	Total			1st semester × 2nd semester	Effect size
Semester							
1st semester	291 (61.8)	180 (38.2)	471				
				4.45	0.03*	1.41 (1.02–1.93)	Small
2nd semester	124 (53.4)	108 (46.6)	232				

Variable	Category type Woman			*X* ^2^	*p*	OR (95% confidence interval)	
	Youth	Senior	Total			1st semester × 2nd semester	Effect size
Semester							
1st semester	346 (64.6)	190 (35.4)	536				
				8.68	0.003*	1.51 (1.15–1.99)	Small
2nd semester	189 (54.6)	157 (45.4)	346				

Source: The authors.

*χ*^2^, Chi-square test; H1, Jan–Jun; H2, Jul–Dec; Effect Size, OR <1.23 (very small), OR between 1.23 and 1.85 (small), OR between 1.86 and 2.99 (medium) and OR > 2.99 (large).

* *p* < 0.05.

**Table 6:** Evaluation of birth semesters of athletes participating in Brazilian national teams between 2002 and 2023: collective performance.

Variable	Collective performance Men			*X* ^2^	*p*	OR (95% confidence interval)	
	Medalist	Non-medalist	Total			1st semester × 2nd semester	Effect size
Semester							
1st semester	262 (55.6)	209 (44.4)	471				
				0.010	0.918	1.02 (0.74–1.40)	Very Small
2nd semester	130 (56.0)	102 (44.0)	232				

Variable	Collective performance Woman			*X* ^2^	*p*	OR (95% confidence interval)	
	Medalist	Non-medalist	Total			1st semester × 2nd semester	Effect size
Semester							
1st semester	368 (68.7)	168 (31.3)	536				
				3.64	0.056	0.76 (0.57–1.008)	Very Small
2nd semester	216 (62.4)	130 (37.6)	346				

Source: The authors.

*χ*^2^, Chi-square test; H1, Jan–Jun; H2, Jul–Dec; Effect Size, OR <1.23 (very small), OR between 1.23 and 1.85 (small), OR between 1.86 and 2.99 (medium) and OR >2.99 (large).

*p* < 0.05.

## Discussion

The aim of the present study was to evaluate the RAE in Brazilian basketball national teams of both genders in official competitions, and to observe its association with competition type, age category and outcome. The findings are similar for both groups; the results indicate that the RAE is present in Brazilian national basketball teams over a 22-year period (2002–2023); in addition, an association between birth semester and type of category. However, no significant differences were observed in the association between birth semesters and competition type or outcome.

The tournaments considered as senior competitions in the sample occur at the end of the developmental stage and in the adult phase (U-17, U-19, and Senior World Cups, and the Olympics), as evidenced by the mean age of this group in the sample (male 25.32 years and female 22.47 years). It is understood that, at this stage, there are indications that the RAE may undergo a reversal, with more individuals born in the second semester (29). A possible reason for this trend can be that maturational and chronological advantages may no longer have as much influence on performance at this stage (13), as shown in the studies by Vegara-Ferri et al. (17) and Werneck et al. (18), which did not find an RAE at the 2012 and 2016 Olympics in either competition (with the exception of the French national team in 2012) and in the investigation by Subijana and Lorenzo (30), where an RAE reversal was observed in the Spanish basketball. Furthermore, those who were born in the second semester and endured and persisted in the selection process, may develop psychological skills that endow these athletes with greater coping ability and resilience to difficulties compared to their peers born in the first months of the year (31).

Although the results of the present study corroborate findings in Brazilian national competitions (20, 21, 23), in the group of individuals who participated in world competitions for the male Brazilian national teams, the highest odds ratio appears in the comparison between Q2 × Q4, with a value considered large (3.18), compared to the results found between Q1 × Q4, with a medium odds ratio (2.62), as well as in the comparison between Q3 × Q4 (2.18). On this basis, it can be inferred that there is a tendency for RAE reversal in this group. In the group of athletes who competed in world competitions for the female Brazilian basketball national teams, the highest odds ratio is between Q1 × Q4 (1.44), which may indeed imply that, for this group, there is a tendency towards a reversal of the phenomenon.

The difference among the number of participants, teams and popularity between the two genders has been associated with a greater presence of RAE in the male gender. In general, when there are more athletes competing for a position, the tendency and magnitude of the effect will be greater (32–34). Additionally, women commonly mature physically earlier, which can interfere in the selection process and, consequently, the RAE (35). However, a systematic review by Baker et al. (36) showed that, at the period of the investigation, only 10% of talent research focused exclusively on a female population, thus, information on this group is scarce. In agreement with the argument above, in an analysis with athletes of both genders who competed in FIBA competitions in the U-14, 15, 16, 17, 18, 20 and senior categories, between 2012 and 2023, the comparison between genders showed a statistical difference, with a higher prevalence of female athletes in the last quartile compared to male athletes (37).

Unlike the tournaments considered senior in the sample, continental tournaments feature competitions with younger athletes, such as the U-15 South American and U-16 America's Cup championships. During this period, maturational and chronological advantages still have a strong influence on individuals' performance, enabling athletes with advanced maturational status, and those born in the early months of the year, to be preferred over their peers who have normative or delayed maturational status and were born in the second semester (10, 11, 38). The findings of the present study corroborate the above, as the RAE was observed in the sample and the highest odds ratios, for both males and females in the group that competed in continental competitions for the Brazilian basketball national teams, are in the comparison between Q1 × Q4 (2.40 for males and 2.19 for females).

Regarding the comparison between birth semesters and competition type (World and Continental), it was not possible to observe a significant association between being born in the first semester and competing in a world competition for the Brazilian basketball national team in either gender. One of the possible reasons for this result could be a larger number of individuals who participated in continental competitions compared to those who competed in world competitions; for males, 559 athletes participated in continental and 144 in world competitions, while for females, 655 athletes competed in continental and 227 in world competitions. This asymmetry is associated with the number of categories contested in continental competitions (7) compared to the world level (4). Moreover, continental competitions qualify teams for world competitions, therefore there are fewer spots available for teams from their respective continents. Thus, national teams generally participate more often in continental than in world competitions. Additionally, the qualification pathway for world competitions means that these tournaments are held with older athletes, and in this period, temporary advantages (maturational and chronological) are no longer determinant for good performance; thus, the lack of association between being born in the first semester of the year and participating in world competitions for the national team seems plausible.

Concerning the age category, the presence of the RAE in the group that competed in youth competitions for the Brazilian basketball national team, regardless of gender, in both the quartile comparison and the association between semester and age category, corroborates the literature (16, 17, 39). Furthermore, during this period, there is greater maturational and biological variability, so athletes in younger categories who possess the aforementioned advantages (chronological, maturational, and biological) tend to be chosen more often (10, 11, 14).

As for the athletes who competed in senior competitions for the male and female Brazilian basketball national teams, the overrepresentation of those born in the first months of the year compared to those born later in the sample, reinforces what was observed in a study that also considered basketball national teams; with 8,664 athletes of both genders, from various categories including seniors, who competed in FIBA championships between 2012 and 2023, where the RAE was observed in each of the categories (37). One possible explanation is the Matthew Effect, in which athletes with early sporting success tend to receive more opportunities, creating a cycle of exclusion for other athletes (40). By being repeatedly selected, an athlete can accumulate advantages in terms of the level and quality of practice, which can lead to performance improvement and, consequently, the exclusion of other athletes.

Nevertheless, in the investigation by Oliveira et al. (14) with athletes from the main men's country competition, the NBB, in the 2014–2015 season, which divided athletes into three career phases within the competition, in development (17–24 years), in consolidation (25–34 years), and final phase (35 years or more), it was possible to observe an RAE reversal in the consolidation and final phases, such that being born in the second semester pointed to greater longevity within the competition. In another analysis, also with NBB athletes, this time in the 2019–2020, 2020–2021, and 2021–2022 seasons, the quartile with the highest number of representatives was the third quartile, indicating an RAE reversal (14, 41).

Accordingly, the reversed RAE has been associated with higher levels of career success in other sports (42, 43). Consequently, the RAE does not seem to be an indicator of career success, as evidenced in the study by Kalén et al. (44), in which, in the national teams of the European continent, only 47.6% of male individuals selected at U-15, were re-selected at U-20, and for females, 52.7% of the athletes chosen at U-15 reached the U-20 level. In addition, those born in the last quarter of the year had a 20% to 25% higher chance of being selected up to age 20, compared to those born in the first quarter of the year. Ribeiro Junior et al. (9) highlight in their investigation that the RAE has a strong influence on elite Brazilian youth championships but is not a determining factor for career success.

Once the results of the competitions considered in the sample were analysed, it was not possible to observe significant differences in the association between semesters and type of outcome. Unlike the present work, in studies that considered this variable for the RAE, it was possible to observe significant differences in the teams that achieved the best placements compared to the teams that occupied the last places; that is, the result suggests that the RAE may be one of the reasons for the better classification of these teams. However, this association of results does not point to the interference of the RAE in the classification of the Brazilian basketball national teams in their respective championships (17, 20, 21).

In the Brazilian context, there is a cultural pressure for immediate results that may contribute to the selection of relatively older individuals (41). On the other hand, even with the potential pressure for immediate results, it is necessary to identify and select those who have a good current performance but also have a good sports projection, taking into consideration not only those who, for momentary reasons, perform better. Once competing at a high level, whether state or national, increases the perception and individual satisfaction of athletes, and consequently increases the chance of reaching high performance. The privilege of competing in high-level competitions may, someway, perpetuate the RAE in Brazilian national teams. Therefore, the findings of this study suggest a better interpretation of the selection and identification process of young athletes, in order to organize a future sporting project for Brazilian basketball (45).

This study presents, for the first time, the RAE in the elite level of Brazilian basketball; i.e., within the Brazilian national basketball teams, and its possible associations over an extended period. However, the fact that the study did not individually track the potential advancement of athletes across categories, which means, it did not identify the factors influencing career progression within the Brazilian national basketball teams, it represents a limitation of the study. Future research able to identify which factors interfere in the career progression within the Brazilian national basketball teams is suggested as a direction for further investigation. This investigation may contribute to improving the understanding of the Relative Age Effect in the process of identifying and selecting young basketball elite players. Nonetheless, it is important to emphasize that such knowledge has little practical value if it does not reach coaches and sport managers. By understanding this phenomenon (RAE), they can promote fairer and more equitable practices during the developmental stages of young basketball players who may, in the future, represent their nation. Categories organized according to maturational stages or the rotating age ranges within the same category, may help minimize the impact of the RAE in basketball. Therefore, understanding the consequences of the RAE can help optimize the time and the resources invested in the sport.

## Conclusion

The findings indicate that the Relative Age Effect (RAE) was present in both men's and women's Brazilian national basketball teams over a 22-year period (2002–2023). An association was observed between the birth semester and the type of age category, suggesting that being born in the first semester of the year increases the young athlete's probability of being selected for youth international competitions in the Brazilian national basketball teams.

However, no significant associations were observed in the relationship between birth semesters, the type of competition, and the results. It implies that being born in the first semester does not appear to increase the young athlete's likelihood of selection for World Championships, nor does it affect the team's overall collective performance. These results suggest a need for improved evaluation in the selection and talent identification process among young players, thus allowing for a better exploration of the full sport potential in Brazil.

## Data Availability

The raw data supporting the conclusions of this article will be made available by the authors, without undue reservation.
